# Exploring the evolution of *Acinetobacter baumannii* and *Pseudomonas aeruginosa* resistance during the COVID-19 era

**DOI:** 10.1017/ash.2025.46

**Published:** 2025-03-26

**Authors:** Emine Sehmen, Esmeray Mutlu Yılmaz, Sevim Yetkin Pusa, Metin Özdemir, Yavuz Yiğit

**Affiliations:** 1 Department of Clinical Microbiolgy and İnfectious Disease, Gazi State Hospital, Samsun, Turkiye; 2 Department of Clinical Microbiolgy and İnfectious Disease, Samsun Training and Research Hospital, Samsun, Turkiye; 3 Department of Emergency Medicine, Hamad Medical Corporation, Doha, Qatar

## Abstract

**Aim:**

In our study, we aim to compare the resistance profiles of *Acinetobacter baumannii* and *Pseudomonas aeruginosa* isolates from intensive care unit (ICU) patients before and during the COVID-19 pandemic.

**Materials:**

The study involved adult patients monitored in the ICUs of a secondary-level hospital from January 2019 to December 2022. Isolates of *A. baumannii* and *P. aeruginosa* were obtained from blood, urine, and respiratory samples. Identification and antibiotic susceptibility tests were conducted using the disk diffusion method and the VITEK 2 system.

**Results:**

The average age of the patients was 61.3 ± 21.9 years (range: 18–95), with a majority of 1306 (51.6%) being male. During the pandemic, *A. baumannii* isolates showed a significant increase in resistance rates for several antibiotics compared to the pre-pandemic period: imipenem (96% vs 35.1%), amikacin (84.1% vs 14.4%), ciprofloxacin (96.9% vs 36.9%), trimethoprim-sulfamethoxazole (66.4% vs 27%), and ceftazidime (96.5% vs 33.3%) (all with *P* < .001). However, there was no significant change in colistin resistance rates in these isolates (0.9% vs 0%; *P* = .307). Similarly, *Pseudomonas aeruginosa* isolates exhibited significant increases in resistance rates during the pandemic compared to the pre-pandemic period: imipenem (51.5% vs 18.8%; *P* < .001), colistin (4.9% vs 0.6%; *P* = .009), amikacin (23.5% vs 4.4%; *P* < .001), ciprofloxacin (53.3% vs 13.8%; *P* < .001), and ceftazidime (39.2% vs 12.7%; *P* < .001).

**Conclusion:**

Our results demonstrate a significant increase in antibiotic resistance levels in *Acinetobacter* and *Pseudomonas* strains associated with hospital-acquired infections or colonization during the COVID-19 pandemic.

## Introduction

Intensive care units (ICUs) are the most common settings for nosocomial infections. Among these infections, clinical presentations can be more severe, and treatment can be particularly challenging when caused by gram-negative bacteria. Infections by non-fermentative gram-negative bacteria, such as *Acinetobacter* and *Pseudomonas* species, offer limited treatment options due to rapidly emerging resistance.^1–3^

The COVID-19 pandemic has profoundly transformed healthcare practices worldwide. As the pandemic unfolded, many routine medical procedures and outpatient services were abruptly halted, and numerous scheduled surgeries were indefinitely postponed or canceled. Consequently, hospital admissions surged, placing significant strain on ICUs (ICUs) as they struggled to manage the influx of COVID-19 patients. Additionally, the increased reliance on antibiotics during this period has led to an alarming rise in antibiotic resistance rates.^4–8^

The escalation in antibiotic resistance, particularly among non-fermentative gram-negative bacteria, has significant implications for patient outcomes in ICUs. The resilience of pathogens such as *Acinetobacter baumannii* and *P. aeruginosa* against multiple antibiotic classes complicates treatment regimens and prolongs hospital stays.^9^ These bacteria possess intrinsic resistance mechanisms and can acquire further resistance through horizontal gene transfer, making them formidable adversaries in clinical settings.^10,11^ The challenge is further exacerbated by the limited pipeline of new antibiotics, underscoring the urgent need for innovative therapeutic strategies and robust infection control measures.^9^

The pandemic’s impact on antibiotic resistance extends beyond the direct effects of increased antibiotic use. Disruptions in healthcare delivery, including reduced infection control oversight, changes in hospital staffing, and the repurposing of healthcare resources, may have inadvertently contributed to the spread of resistant strains.^12,13^ Additionally, the overuse and misuse of antibiotics in both COVID-19 and non-COVID-19 patients during the pandemic may have accelerated resistance development.^14^

Our study aims to compare the resistance profiles of *A. baumannii* and *P. aeruginosa* isolates from ICU patients before and during the COVID-19 pandemic to assess the impact of the pandemic on antibiotic resistance.

## Materials and methods

### Methods

#### Study design and setting

This retrospective descriptive study was conducted at Gazi State Hospital, a secondary-level general hospital located in Samsun, a major city in northern Turkey with a population of 1.3 million. The hospital has a bed capacity of 350, including 62 ICU beds. Ethical approval was obtained from the Samsun University Clinical Research Ethics Committee (decision number SÜKAEK-2023 20/21), and the study adhered to the principles of the Declaration of Helsinki and Good Clinical Practice guidelines. Given the retrospective nature of the study, the ethics committee waived the requirement for informed consent. For the purpose of this study, the pre-pandemic period was defined as January to December 2019, while the pandemic period was defined as January 2020 onwards. This classification aligns with the emergence of COVID-19 in the country, where the first reported cases occurred in 2020. This approach allows a focused analysis of resistance patterns associated with the pandemic and its immediate impact.

#### Patient selection

The study included adult patients (≥ 18 years) admitted to ICUs for various medical reasons between January 2019 and December 2022. Inclusion criteria encompassed the presence of *A. baumannii* and *P. aeruginosa* identified in cultures obtained from blood, urine, respiratory, wound, and intravenous catheter samples of ICU patients. While all samples with microbial growth were analyzed, no positive bone cultures were identified during the study period, which is why these were not included in the manuscript. The discussion primarily focuses on blood, urine, and respiratory samples, as they accounted for the majority of significant infections in ICU settings and are central to the study’s objectives of analyzing systemic infections and their resistance patterns. For patients with multiple positive cultures, only the initial culture data were analyzed. Bacterial species other than *A. baumannii* and *P. aeruginosa* were excluded from the study.

#### Culture collection and microbial ıdentification

Cultures were obtained from patients using standard aseptic techniques. Blood cultures were collected using sterile needles and syringes, with blood samples immediately inoculated into aerobic and anaerobic culture bottles. Urine samples were collected using sterile containers, and respiratory samples were obtained via tracheal aspirates or bronchoalveolar lavage using sterile techniques.

All positive cultures were subjected to microbial identification using both traditional biochemical methods and the automated VITEK 2 system (bioMérieux, France). Traditional methods included Gram staining, oxidase tests, and biochemical tests for specific enzyme activities and carbohydrate utilization patterns.

#### Antibiotic susceptibility testing

Antibiotic susceptibility testing was conducted on all isolates using the disk diffusion method and the VITEK 2 system. Disk diffusion tests were performed on Mueller-Hinton agar plates, where antibiotic-impregnated disks were placed on the inoculated agar surface. The plates were incubated at 35°C for 16–18 hours, and the zone diameters were measured to determine susceptibility according to Clinical and Laboratory Standards Institute (CLSI) criteria.^15^

The VITEK 2 system provided automated identification and susceptibility results by analyzing microbial growth patterns in the presence of various antibiotics. Retrospective sensitivity data were retrieved from the hospital’s electronic medical records and the VITEK 2 system’s database, ensuring compliance with CLSI standards.

### Statistical analysis

All statistical analyses in the study were conducted using IBM SPSS Statistics for Windows, Version 25.0 (IBM Corp., Armonk, NY, USA). Descriptive data were reported as frequencies and percentages to summarize the characteristics of the study population. For comparisons between groups involving categorical variables, Pearson’s χ^2^ test was employed to determine the presence of any statistically significant associations. The significance level for all tests was set at a 95% confidence interval, with *P*-values less than .05 considered indicative of statistical significance. Additionally, where multiple comparisons were made, the Bonferroni correction was applied to adjust for the increased risk of type I errors, ensuring that the reported *P*-values remained robust and reliable.

## Results

The mean age of the participants was 61.3 ± 21.9 years (range: 18–95), with males comprising 51.6% of the total population. The distribution of culture findings is detailed in Table 1.

**Table 1.** Distribution of the cultures

		Urine n (%)	Blood n (%)	Tracheal aspirate-sputum n (%)	Wound n (%)	IV Catheter n (%)	Total n (%)
General	*Escherichia coli*	592 (41)	47 (6.8)	86 (8.7)	92 (25.4)	3 (6.4)	820 (23.2)
	*Pseudomonas aeruginosa*	217 (15)	84 (12.2)	263 (26.6)	58 (16)	11 (23.4)	633 (17.9)
	*Acinetobacter baumannii*	70 (4.8)	98 (14.2)	320 (32.4)	41 (11.3)	11 (23.4)	540 (15.3)
	*Klebsiella* spp.	180 (12.5)	144 (20.8)	152 (15.4)	38 (10.5)	9 (19.1)	523 (14.8)
	*Enterococcus* spp.	177 (12.3)	163 (23.6)	9 (0.9)	41 (11.3)	6 (12.8)	396 (11.2)
	*Staphylococcus aureus*	24 (1.7)	71 (10.3)	96 (9.7)	58 (16)	1 (2.1)	250 (7.1)
	*Proteus* spp.	82 (5.7)	27 (3.9)	20 (2)	34 (9.4)	3 (6.4)	166 (4.7)
	*Candida* spp.	34 (2.4)	46 (6.7)	8 (0.8)	0 (0)	2 (4.3)	90 (2.5)
	*Stenotrophomonas maltophilia*	2 (0.1)	9 (1.3)	32 (3.2)	0 (0)	1 (2.1)	44 (1.2)
	*Providencia* spp.	34 (2.4)	2 (0.3)	3 (0.3)	0 (0)	0 (0)	39 (1.1)
	*Myroides* spp.	32 (2.2)	0 (0)	0 (0)	0 (0)	0 (0)	32 (0.9)
	Total	1444 (100)	691 (100)	989 (100)	362 (100)	47 (100)	3533 (100)
2019	*Pseudomonas aeruginosa*	64 (20.8)	25 (10.7)	70 (38.3)	20 (16.9)	2 (22.2)	181 (21.3)
	*Escherichia coli*	115 (37.3)	18 (7.7)	9 (4.9)	24 (20.3)	0 (0)	166 (19.5)
	*Klebsiella* spp.	44 (14.3)	42 (18)	29 (15.8)	15 (12.7)	4 (44.4)	134 (15.7)
	*Enterococcus* spp.	37 (12)	62 (26.6)	1 (0.5)	15 (12.7)	2 (22.2)	117 (13.7)
	*Acinetobacter baumannii*	16 (5.2)	24 (10.3)	49 (26.8)	21 (17.8)	1 (11.1)	111 (13)
	*Staphylococcus aureus*	5 (1.6)	22 (9.4)	14 (7.7)	11 (9.3)	0 (0)	52 (6.1)
	*Candida* spp.	6 (1.9)	29 (12.4)	2 (1.1)	0 (0)	0 (0)	37 (4.3)
	*Proteus* spp.	13 (4.2)	7 (3)	3 (1.6)	12 (10.2)	0 (0)	35 (4.1)
	*Stenotrophomonas maltophilia*	0 (0)	2 (0.9)	6 (3.3)	0 (0)	0 (0)	8 (0.9)
	*Providencia* spp.	3 (1)	2 (0.9)	0 (0)	0 (0)	0 (0)	5 (0.6)
	*Myroides* spp.	5 (1.6)	0 (0)	0 (0)	0 (0)	0 (0)	5 (0.6)
	Total	308 (100)	233 (100)	183 (100)	118 (100)	9 (100)	851 (100)
2020	*Escherichia coli*	132 (37.7)	5 (3.7)	21 (10.2)	17 (25)	0 (0)	175 (22.8)
	*Pseudomonas aeruginosa*	67 (19.1)	30 (22.2)	44 (21.5)	12 (17.6)	1 (12.5)	154 (20.1)
	*Acinetobacter baumannii*	13 (3.7)	14 (10.4)	69 (33.7)	6 (8.8)	1 (12.5)	103 (13.4)
	*Klebsiella* spp.	43 (12.3)	26 (19.3)	21 (10.2)	3 (4.4)	0 (0)	93 (12.1)
	*Enterococcus* spp.	40 (11.4)	26 (19.3)	3 (1.5)	11 (16.2)	2 (25)	82 (10.7)
	*Staphylococcus aureus*	6 (1.7)	16 (11.9)	33 (16.1)	15 (22.1)	1 (12.5)	71 (9.3)
	*Proteus* spp.	28 (8)	7 (5.2)	5 (2.4)	4 (5.9)	1 (12.5)	45 (5.9)
	*Candida* spp.	8 (2.3)	6 (4.4)	2 (1)	0 (0)	1 (12.5)	17 (2.2)
	*Stenotrophomonas maltophilia*	1 (0.3)	5 (3.7)	7 (3.4)	0 (0)	1 (12.5)	14 (1.8)
	*Providencia* spp.	9 (2.6)	0 (0)	0 (0)	0 (0)	0 (0)	9 (1.2)
	*Myroides* spp.	3 (0.9)	0 (0)	0 (0)	0 (0)	0 (0)	3 (0.4)
	Total	350 (100)	135 (100)	205 (100)	68 (100)	8 (100)	766 (100)
2021	*Escherichia coli*	147 (46.5)	13 (6.7)	18 (9.5)	22 (27.5)	1 (7.1)	201 (25.4)
	*Acinetobacter baumannii*	17 (5.4)	37 (19.2)	89 (47.1)	5 (6.3)	6 (42.9)	154 (19.4)
	*Klebsiella* spp.	39 (12.3)	39 (20.2)	16 (8.5)	10 (12.5)	1 (7.1)	105 (13.3)
	*Pseudomonas aeruginosa*	31 (9.8)	22 (11.4)	35 (18.5)	12 (15)	4 (28.6)	104 (13.1)
	*Enterococcus* spp.	41 (13)	48 (24.9)	2 (1.1)	6 (7.5)	1 (7.1)	98 (12.4)
	*Staphylococcus aureus*	4 (1.3)	17 (8.8)	13 (6.9)	16 (20)	0 (0)	50 (6.3)
	*Proteus* spp.	15 (4.7)	9 (4.7)	8 (4.2)	9 (11.3)	0 (0)	41 (5.2)
	*Candida* spp.	7 (2.2)	7 (3.6)	1 (0.5)	0 (0)	1 (7.1)	16 (2)
	*Myroides* spp.	10 (3.2)	0 (0)	0 (0)	0 (0)	0 (0)	10 (1.3)
	*Providencia* spp.	5 (1.6)	0 (0)	2 (1.1)	0 (0)	0 (0)	7 (0.9)
	*Stenotrophomonas maltophilia*	0 (0)	1 (0.5)	5 (2.6)	0 (0)	0 (0)	6 (0.8)
	Total	316 (100)	193 (100)	189 (100)	80 (100)	14 (100)	792 (100)
2022	*Escherichia coli*	198 (42.1)	11 (8.5)	38 (9.2)	29 (30.2)	2 (12.5)	278 (24.7)
	*Pseudomonas aeruginosa*	55 (11.7)	7 (5.4)	114 (27.7)	14 (14.6)	4 (25)	194 (17.3)
	*Klebsiella* spp.	54 (11.5)	37 (28.5)	86 (20.9)	10 (10.4)	4 (25)	191 (17)
	*Acinetobacter baumannii*	24 (5.1)	23 (17.7)	113 (27.4)	9 (9.4)	3 (18.8)	172 (15.3)
	*Enterococcus* spp.	59 (12.6)	27 (20.8)	3 (0.7)	9 (9.4)	1 (6.3)	99 (8.8)
	*Staphylococcus aureus*	9 (1.9)	16 (12.3)	36 (8.7)	16 (16.7)	0 (0)	77 (6.9)
	*Proteus* spp.	26 (5.5)	4 (3.1)	4 (1)	9 (9.4)	2 (12.5)	45 (4)
	*Candida* spp.	13 (2.8)	4 (3.1)	3 (0.7)	0 (0)	0 (0)	20 (1.8)
	*Providencia* spp.	17 (3.6)	0 (0)	1 (0.2)	0 (0)	0 (0)	18 (1.6)
	*Stenotrophomonas maltophilia*	1 (0.2)	1 (0.8)	14 (3.4)	0 (0)	0 (0)	16 (1.4)
	*Myroides* spp.	14 (3)	0 (0)	0 (0)	0 (0)	0 (0)	14 (1.2)
	Total	470 (100)	130 (100)	412 (100)	96 (100)	16 (100)	1124 (100)

Spp, severel species; IV, ıntravenous.

**Figure 1.**
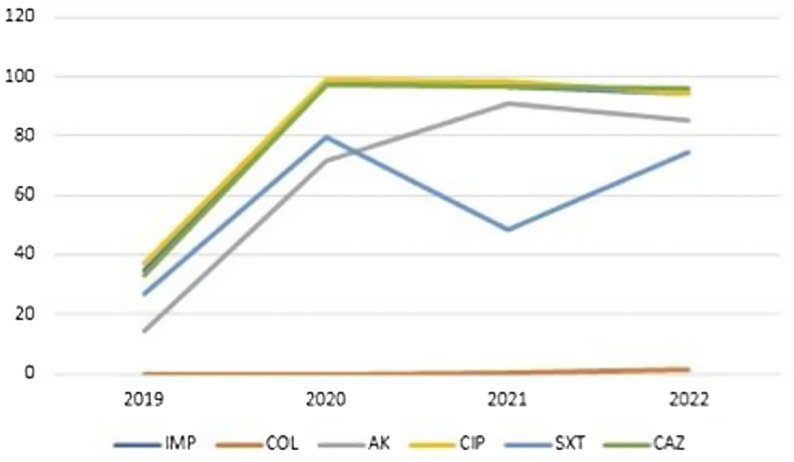
Change in resistance rates througout the years in *Acinetobacter baumanni* isolates.

A significant increase in resistance rates was observed in *A. baumannii* isolates during the COVID-19 pandemic compared to the pre-pandemic period, particularly for imipenem (96% vs 35.1%), amikacin (84.1% vs 14.4%), ciprofloxacin (96.9% vs 36.9%), trimethoprim-sulfamethoxazole (66.4% vs 27%), and ceftazidime (96.5% vs 33.3%) (Figure 1). However, there was no significant change in resistance rates to colistin (0.9% vs 0%; *P* = .307) (Table 2).

**Table 2.** Resistance rates in non-fermantary bacteria

	2019		2020–2022		*P* ^*^	2020		2021		2022		P^**^
	n	%	n	%		n	%	n	%	n	%	
*Acinetobacter baumanniin*	111		429			103		154		172		
IMP	39	35.1	412	96	**<.001**	101	98.1	149	96.8	162	94.2	**<.001**
COL	0	0	4	0.9	.307	0	0	1	0.6	3	1.7	.265
AK	16	14.4	361	84.1	**<.001**	74	71.8	140	90.9	147	85.5	**<.001**
CIP	41	36.9	415	96.7	**<.001**	102	99.0	151	98.1	162	94.2	**<.001**
SXT	30	27.0	285	66.4	**<.001**	82	79.6	75	48.7	128	74.4	**<.001**
CAZ	37	33.3	414	96.5	**<.001**	100	97.1	149	96.8	165	95.9	**<.001**
*Pseuomonas aeruginosan*	181		452			154		104		194		
IMP	34	18.8	233	51.5	**<.001**	74	48.1	37	35.6	122	62.9	**<.001**
COL	1	0.6	22	4.9	**.009**	8	5.2	6	5.8	8	4.1	.058
AK	8	4.4	106	23.5	**<.001**	28	18.2	19	18.3	59	30.4	**<.001**
CIP	25	13.8	241	53.3	**<.001**	76	49.4	43	41.3	122	62.9	**<.001**
CAZ	23	12.7	177	39.2	**<.001**	42	27.3	38	36.5	97	50.0	**<.001**

* The comparison between 2019 and 2020–2022,

** The comparison among 2019, 2020, 2021 and 2022. IPM, Imipenem; COL, Colistin; AMP, Ampicillin; AK, Amikacin; CIP, Ciprofloxacin; SXT, Trimethoprim\sulfamethoxazole; CAZ, Ceftazidime.

*P. aeruginosa* isolates also demonstrated a significant increase in resistance rates during the pandemic compared to the pre-pandemic period for imipenem (51.5% vs 18.8%; *P* < .001), colistin (4.9% vs 0.6%; *P* = .009), amikacin (23.5% vs 4.4%; *P* < .001), ciprofloxacin (53.3% vs 13.8%; *P* < .001), and ceftazidime (39.2% vs 12.7%; *P* < .001).

**Figure 2.**
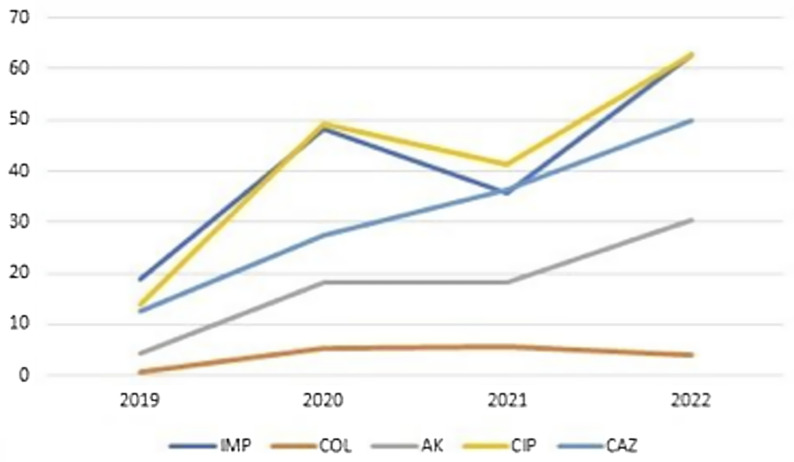
Change in resistance rates througout the years in *Pseudomonas aeruginosa* isolates.

Pairwise comparisons across the years indicated that resistance rates for both bacteria were significantly lower in 2019 compared to subsequent years. Furthermore, no statistically significant changes in resistance rates were observed between 2020 and 2022 (*P* > .05) (Table 2) (Figure 2).

Figures 1 and 2 visually illustrate the trends in resistance rates for *A. baumannii* and *P. aeruginosa*, respectively, as also detailed in Table 2.

## Discussion

The COVID-19 pandemic has been associated with a significant increase in *Acinetobacter*-related hospital infections.^16^ This surge can be attributed to several factors, including higher admissions to ICUs, prolonged hospital stays, increased utilization and duration of mechanical ventilation, and oxygen support, particularly in patients with COVID-19.^16^ Reports indicate a global rise in antibiotic resistance rates among all bacterial species responsible for hospital infections, driven by bacterial superinfections in hospitalized patients during the pandemic.^17,18^ A meta-analysis has demonstrated that the resistance rates of gram-negative bacteria have increased during the COVID-19 pandemic compared to pre-pandemic levels. Specifically, *Acinetobacter* isolates have shown significant increases in resistance rates for amikacin, ceftazidime, imipenem, and ciprofloxacin.^18^ Raoufi et al^19^ also reported elevated resistance rates for ciprofloxacin, gentamicin, ceftriaxone, and cefotaxime in *A. baumannii* isolates during the pandemic compared to pre-pandemic levels. Furthermore, a study by Pascale et al^20^ revealed a substantial rise in carbapenem-resistant *A. baumannii* rates even in the early months of the pandemic. Our study corroborates these findings, uncovering a significant increase in resistance rates for imipenem (96% vs 35.1%), amikacin (84.1% vs 14.4%), ciprofloxacin (96.9% vs 36.9%), trimethoprim-sulfamethoxazole (66.4% vs 27.0%), and ceftazidime (96.5% vs 33.3%) in *A. baumannii* isolates during the pandemic compared to pre-pandemic levels. These results highlight a considerable escalation in resistance rates across various antibiotic groups in *Acinetobacter* isolates during the pandemic, underscoring the limited treatment options available for *Acinetobacter*-related hospital infections in this context. Year-to-year comparisons indicated that resistance rates were significantly lower in 2019 compared to subsequent years, with no substantial change observed between 2020 and 2022. This suggests that the notable surge in resistance rates occurred at the onset of the pandemic but did not persist throughout its duration. Contrary to the typical upward trend in resistance rates over time, this phenomenon implies a direct impact of the pandemic on bacterial resistance patterns.

*P. aeruginosa* is a non-fermentative gram-negative bacterium that frequently causes severe and resistant infections, particularly in ICU settings.^18,21^ Serretiello et al^22^ reported a significant increase in resistance rates for antibiotics other than imipenem among *P. aeruginosa* isolates obtained during the pandemic. Similarly, a multicenter study conducted in Mexico observed increased resistance rates to imipenem and ciprofloxacin in *P. aeruginosa* isolates during the pandemic compared to the pre-pandemic period.^23^ In a study conducted in Kuwait, Alali et al^21^ found that resistance rates to gentamicin in *P. aeruginosa* isolates obtained from hospital wards increased during the pandemic compared to the pre-pandemic period, while resistance rates to amikacin, ceftazidime, and meropenem showed no significant changes. Conversely, Coserio et al^18^ reported no significant changes in resistance rates to imipenem, levofloxacin, amikacin, and ceftazidime during the pandemic in *P. aeruginosa* isolates. Similarly, Despotovic et al^24^ found no significant changes in resistance rates to cephalosporins, aminoglycosides, fluoroquinolones, or carbapenems in *P. aeruginosa* isolates during the pandemic. However, Raoufi et al^25^ reported increased resistance rates to ciprofloxacin, gentamicin, and cefepime in *P. aeruginosa* isolates during the pandemic compared to the pre-pandemic period. Mena-Lora et al^26^ also demonstrated an increase in carbapenem resistance rates among *P. aeruginosa* isolates during the pandemic.

In our study, significant increases in resistance rates were observed in *P. aeruginosa* isolates during the pandemic compared to the pre-pandemic period, including imipenem (51.5% vs 18.8%), colistin (4.9% vs 0.6%), amikacin (23.5% vs 4.4%), ciprofloxacin (53.3% vs 13.8%), and ceftazidime (39.2% vs 12.7%). These findings indicate that *Pseudomonas* isolates became generally more resistant to antibiotics during the pandemic, significantly limiting treatment options for hospital-acquired infections caused by *P. aeruginosa*.

Our study used data from January 2019 as the pre-pandemic baseline and data from January 2020 onward to represent the pandemic period. The division was based on the time line of COVID-19 emergence in the country, with the first confirmed cases in March 2020. While a longer pre-pandemic observation period might provide broader insights into resistance trends over time, this study specifically aimed to evaluate the pandemic’s immediate impact. This focused time frame ensures that the findings are relevant to the healthcare challenges encountered during the COVID-19 pandemic. All sample types with microbial growth were analyzed, as detailed in the methodology, and wound and intravenous catheter samples were included. However, no positive bone cultures were observed during the study period, and the discussion centered on blood, urine, and respiratory samples, which represented the majority of significant ICU infections.

During the pandemic period, the high demand in ICUs necessitated the rotation of physicians from various specialties. Consequently, rational antibiotic practices often fell outside the control of infectious disease specialists. Broad-spectrum antibiotics were initiated as the first-line treatment option and could not be de-escalated. We hypothesize that this is a primary factor contributing to the development of resistance. This situation can significantly accelerate the emergence of antibiotic resistance. The extensive use of broad-spectrum antibiotics during crisis periods facilitates the development of resistance among microorganisms. Revisiting rational antibiotic use and implementing stringent infection control strategies are crucial in mitigating this resistance. Emerging diagnostic biomarkers, such as procalcitonin, may offer potential in guiding antibiotic stewardship during such crises.^27^ Restoring specialist oversight in antibiotic selection and utilization is essential to curb the progression of antibiotic resistance.

This study has several limitations. First, while it provides valuable insights into resistance patterns of *A. baumannii* and *P. aeruginosa* during the COVID-19 pandemic, it does not include data on the specific antibiotics administered to ICU patients. Increased antibiotic usage is a plausible explanation for the rise in resistance rates observed during the pandemic. However, analyzing antibiotic usage data was beyond the scope of this study, as it would have significantly increased its complexity and shifted the focus away from the primary objective. Investigating the relationship between antibiotic prescribing practices and resistance trends is an important topic that warrants a dedicated study. Second, molecular typing or genomic analysis of isolates was not conducted, which limits the ability to identify specific epidemic strains or resistance mechanisms. Third, the study was conducted in a single hospital, which may limit the generalizability of the findings to other healthcare settings with different epidemiological profiles. Finally, while all available clinical samples with microbial growth were analyzed, no positive results were observed for bone cultures, which may have led to underrepresentation of certain infection types. Despite these limitations, the study provides critical insights into the evolving resistance patterns of two key nosocomial pathogens during the pandemic, emphasizing the urgent need for enhanced antibiotic stewardship and infection control measures.

Our results indicate a significant increase in resistance levels in *Acinetobacter* and *Pseudomonas* strains during the pandemic. This finding underscores the increased challenges in managing infections caused by these bacteria due to heightened resistance, highlighting the critical need for thorough monitoring and judicious antibiotic use in response to evolving healthcare circumstances. Nosocomial infections caused by non-fermentative gram-negative bacteria have limited treatment options, emphasizing the importance of closely monitoring resistance patterns. Reports during the COVID-19 pandemic indicate an increase in resistance rates among these bacteria.

## Conclusion

In conclusion, our study has revealed a significant and concerning increase in resistance rates to multiple critical antibiotic groups in *Acinetobacter* and *Pseudomonas* isolates responsible for hospital-acquired infections or colonization during the COVID-19 pandemic. This underscores the urgent need for enhanced antibiotic stewardship and rigorous infection control measures to combat the growing threat of antibiotic resistance in healthcare settings.

## Data Availability

All data and materials from the study are available from the corresponding author upon reasonable request.
